# Mesenteric Steal Syndrome Caused by Abdominal Aortic Stenosis due to Takayasu Arteritis

**DOI:** 10.70352/scrj.cr.25-0631

**Published:** 2026-02-06

**Authors:** Shoichiro Nagashima, Kentaro Inoue, Yusuke Fujioka, Kohei Ueno, Go Kinoshita, Shinichiro Yoshino, Koichi Morisaki, Tomoharu Yoshizumi

**Affiliations:** Department of Surgery and Science, Graduate School of Medical Sciences, Kyushu University, Fukuoka, Fukuoka, Japan

**Keywords:** chronic mesenteric ischemia, mesenteric steal syndrome, Takayasu arteritis

## Abstract

**INTRODUCTION:**

Chronic mesenteric ischemia (CMI) is usually caused by atherosclerotic stenosis of multiple mesenteric arteries. However, aortic stenosis secondary to Takayasu arteritis can rarely cause intestinal ischemia through hemodynamic steal from the mesenteric to lower limb circulation.

**CASE PRESENTATION:**

A 67-year-old woman with a history of Takayasu arteritis presented with postprandial and exertional abdominal pain and a 3-kg weight loss over 2 weeks. CTA revealed isolated severe stenosis of the infrarenal aorta with development of the arc of Riolan, and Doppler ultrasonography demonstrated retrograde flow in the inferior mesenteric artery. Based on these findings, a diagnosis of mesenteric steal syndrome secondary to Takayasu arteritis was made. Endovascular stenting of the infrarenal aorta was performed, resulting in improved antegrade aortic flow and reduced collateral circulation. After treatment, the patient’s abdominal pain resolved and her weight recovered.

**CONCLUSIONS:**

This rare case demonstrates that Takayasu arteritis can cause mesenteric steal syndrome without mesenteric arterial lesions and highlights that endovascular stenting can effectively restore physiological blood flow and relieve ischemic symptoms.

## INTRODUCTION

Chronic mesenteric ischemia (CMI) develops mainly because of chronic atherosclerosis within the mesenteric vessels. The bowel receives its major vascular supply from the celiac artery (CA), superior mesenteric artery (SMA), and inferior mesenteric artery (IMA). These vessels form collateral circulation, such as the marginal artery of Drummond and the arc of Riolan, and symptomatic mesenteric ischemia is often caused by severe stenosis or occlusion of more than two of the three vessels.^1)^ Takayasu arteritis is a chronic large-vessel vasculitis that predominantly affects the aorta and its major branches.^2)^ Inflammatory changes of the arterial wall followed by fibrosis can result in segmental or focal stenosis, occlusion, or, less commonly, aneurysm formation.^3)^ Although aortic involvement in Takayasu arteritis is often associated with aneurysmal change or dissection, focal aortic stenosis can also occur and may lead to clinically significant ischemic symptoms through hemodynamic alterations.^2)^

We report a rare case of CMI caused by stenosis of the infrarenal aorta due to Takayasu arteritis. The patient experienced intestinal ischemic symptoms induced by increased blood flow from the intestine to the lower extremities, which is called mesenteric steal syndrome.

## CASE PRESENTATION

**Fig. 1:**
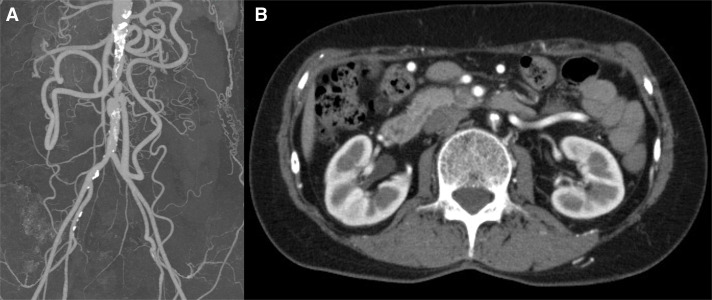
Preprocedural CTA. (**A**) CTA showing severe stenosis of the aorta between the renal arteries and the IMA, and well-developed collateral flow to the distal abdominal aorta. (**B**) Axial image of the stenosis in the infrarenal abdominal aorta. IMA, inferior mesenteric artery

**Fig. 2:**
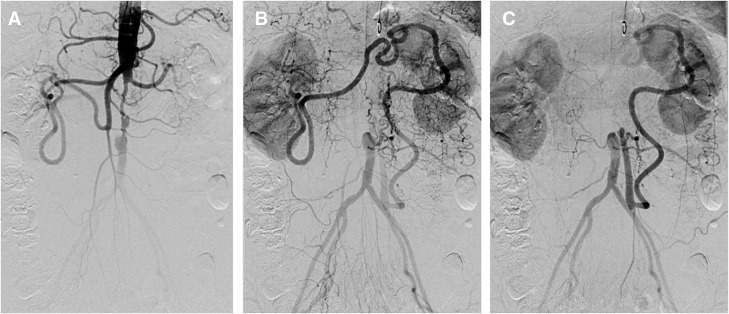
Preprocedural angiography. (**A**) Early-phase antegrade blood flow to the distal aorta via the abdominal aorta. (**B**) Delayed filling of the arc of Riolan via the superior mesenteric artery. (**C**) Late-phase retrograde blood flow in the inferior mesenteric artery, which supplied blood flow to the distal abdominal aorta.

A 67-year-old woman presented with a 2-week history of progressive and intermittent postprandial abdominal pain and weight loss of 3 kg. Her abdominal pain was also provoked by eating, standing, and ambulation. She had a history of Takayasu arteritis, with stenosis of the infrarenal aorta. The patient was diagnosed with Takayasu arteritis at the age of 32, presenting with fever and intermittent claudication. At the time of diagnosis, imaging studies revealed stenosis of the abdominal aorta, and lung perfusion scintigraphy demonstrated a perfusion defect in the right lower lobe. Other possible diseases were excluded, leading to a definitive diagnosis of Takayasu arteritis. No significant carotid artery stenosis or ocular ischemic findings were detected. During long-term follow-up, neither severe atherosclerotic changes nor arterial dissection developed. At disease onset, inflammatory markers were only mildly elevated, and the patient was managed conservatively with warfarin anticoagulation alone, without corticosteroid therapy. At the current presentation, no evidence of clinically significant active vascular lesions was identified outside the abdominal aorta. Upon physical examination, the abdomen was soft and nondistended. Femoral pulses were weakly palpable bilaterally, with nonpalpable popliteal and pedal pulses. The ankle–brachial index of the right and left extremities was 0.64 and 0.65, respectively. During abdominal ultrasonography, retrograde blood flow from the IMA to the abdominal aorta was observed, with a blood flow velocity of 101.6 cm/s. Endoscopic evaluation showed no remarkable findings. CTA showed marked isolated short-segment severe stenosis of the aorta between the renal arteries and the IMA. The SMA and IMA were connected through the developed arc of Riolan. There were no findings of blood flow disturbance in the CA or SMA (**Fig. 1**). Biphasic blood flow below the IMA, direct flow from the aorta, followed by collateral flow via the arc of Riolan and IMA, were observed with diagnostic angiography (**Fig. 2**). To assess potential relapse of arteritis, additional examinations, including FDG-PET, were conducted and revealed no evidence of active inflammatory uptake in the aortic wall.

Considering the abdominal angina induced by eating or ambulation, and retrograde collateral flow from the IMA via the arc of Riolan, we diagnosed mesenteric steal syndrome. Consequently, percutaneous angioplasty was planned to reduce the collateral flow from the IMA.

**Fig. 3:**
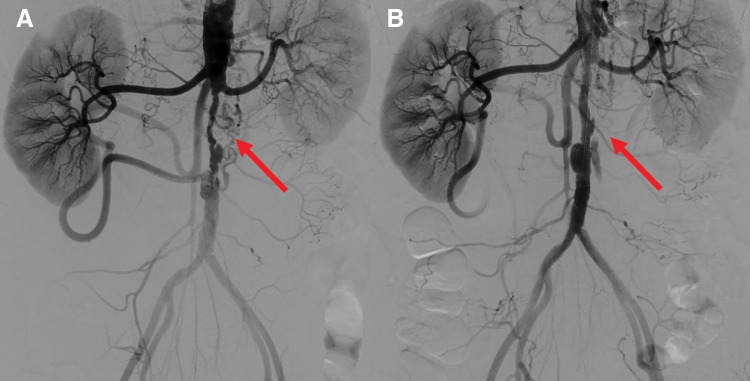
Abdominal aortography before and after treatment. (**A**) Severe stenosis of the infrarenal aorta before stent placement (the red arrow). (**B**) Postprocedural aortography showing dilation of the infrarenal abdominal aorta and increased antegrade blood flow.

Under local anesthesia, sheaths were inserted percutaneously via the left brachial artery and right common femoral artery. Angiography was performed with a pigtail catheter via the left brachial artery to evaluate the aortic lesion. A Radifocus (Terumo, Japan) guidewire and straight catheter were inserted from the right sheath to the terminal aorta. Angiography demonstrated severe focal stenosis of the infrarenal aorta, with a minimal luminal diameter of approximately 3 mm. In addition, the abdominal aorta was diffusely small in caliber, measuring approximately 10 mm even in the distal segment. The pressure gradient between the suprarenal aorta and terminal aorta was 60 mmHg. Based on these anatomical findings, we considered that securing an aortic lumen of approximately 6–7 mm would be sufficient to restore adequate antegrade blood flow while minimizing excessive stress on the vessel wall. Therefore, an 8-mm self-expanding bare-metal stent was selected, followed by post-dilation with a 6-mm balloon. First, the lesion was gently dilated with a 4 × 40 mm SABER X balloon (Cordis, Santa Clara, CA, USA), and a S.M.A.R.T. 8 mm × 6 cm stent (Cordis) was deployed. Post-ballooning was performed with a Sterling 6 × 40 mm (Boston Scientific, Marlborough, MA, USA) balloon. Postprocedural angiography showed improved blood flow in the infrarenal aorta and a decrease in the pressure gradient from 60 to 30 mmHg (**Fig. 3**).

The patient’s postoperative clinical course was favorable. After endovascular treatment, dual antithrombotic therapy with clopidogrel 75 mg once daily and rivaroxaban 2.5 mg twice daily (total daily dose, 5 mg) was initiated to prevent stent thrombosis and distal embolic events. Her abdominal angina resolved, and her weight had increased by 3 kg, 3 months after treatment. After stent placement, the ankle–brachial index improved to 0.80 on the right and 0.77 on the left. Follow-up abdominal ultrasonography showed that retrograde flow from the IMA had decreased markedly to 27.3 cm/s.

## DISCUSSION

CMI is a rare disease. According to the annual report of National Clinical Database, among 105468 cases of peripheral vascular interventions in Japan in 2023, only 21 cases (0.02%) involved endovascular treatment for the mesenteric artery.^4)^ Then, CMI caused by a solitary lesion within the abdominal aorta is extremely rare. In a PubMed search, only one case of CMI was reported with abdominal angina caused by aortic stenosis and reduced blood flow after exercise.^5)^ Mirza et al. reported a decline in mesenteric blood perfusion due to localized stenosis in the aorta between the renal arteries and the IMA.^5)^ In that case, localized stenosis between the SMA and the IMA resulted in the development of the arc of Riolan. The authors hypothesized that an increase in intestinal blood flow demand due to not only a meal but also a decrease in intestinal blood supply owing to lower limb exercise, can lead to symptoms of intestinal ischemia. The authors named this disorder “mesenteric steal syndrome.” Our case is identical to that of Mirza et al. regarding the clinical course and anatomical location of the vascular lesion. With respect to treatment, we performed aortic stenting to increase blood flow in the main abdominal aorta and decrease dependency on collateral circulation pathways. There were two reasons for selecting a self-expanding bare-metal stent and not a balloon-expandable stent or a stent-graft. First, considering that the aortic stenosis was caused mainly by Takayasu arteritis rather than atherosclerosis, using a balloon-expandable stent carried the risk of vascular injury. Compared with atherosclerotic lesions, Takayasu arteritis causes arterial wall scarring and fibrosis, which makes vessels fragile.^6,7)^ Therefore, a self-expanding bare-metal stent, which imposes less stress on the vessel wall, was chosen.^6,8)^ Second, the use of self-expanding bare-metal stents could preserve collateral circulation pathways. Preserving collateral circulation is important to prevent lower limb ischemia caused by stent occlusion. Our challenging treatment was performed effectively and safely, in this case. Treatment resolved the patient’s abdominal angina, which was induced by food intake and ambulation. This suggests that reducing collateral flow via the arc of Riolan can be sufficient to treat mesenteric steal syndrome.

## CONCLUSIONS

CMI without lesions in the CA and/or SMA is extremely rare. Obtaining a careful history and appropriate evaluation of the relationship between symptoms and intestinal blood flow are necessary for effective treatment of mesenteric steal syndrome.

## DECLARATIONS

### Availability of data and materials

All data generated or analyzed during this study are included in this published article.
